# The Neurotoxicity of Nitrous Oxide: The Facts and “Putative” Mechanisms

**DOI:** 10.3390/brainsci4010073

**Published:** 2014-01-28

**Authors:** Sinead Savage, Daqing Ma

**Affiliations:** Anaesthetics, Pain Medicine & Intensive Care, Department of Surgery & Cancer, Imperial College London, Chelsea and Westminster Hospital, London SW10 9NH, UK; E-Mail: s.savage@imperial.ac.uk

**Keywords:** nitrous oxide, neurotoxicity, homocysteine, NMDA antagonist

## Abstract

Nitrous oxide is a widely used analgesic agent, used also in combination with anaesthetics during surgery. Recent research has raised concerns about possible neurotoxicity of nitrous oxide, particularly in the developing brain. Nitrous oxide is an *N*-methyl-d-aspartate (NMDA)-antagonist drug, similar in nature to ketamine, another anaesthetic agent. It has been linked to post-operative cardiovascular problems in clinical studies. It is also widely known that exposure to nitrous oxide during surgery results in elevated homocysteine levels in many patients, but very little work has investigated the long term effect of these increased homocysteine levels. Now research in rodent models has found that homocysteine can be linked to neuronal death and possibly even cognitive deficits. This review aims to examine the current knowledge of mechanisms of action of nitrous oxide, and to describe some pathways by which it may have neurotoxic effects.

## 1. Introduction

Nitrous oxide (N_2_O) has been used alone or in combination with other agents to produce analgesia and anaesthesia for over 150 years [1]. It shows anaesthetic properties at very high concentrations, with a minimum alveolar concentration needed to produce anaesthesia in 50% of subjects (MAC) of 104% [2]. To reach near this level without compromising oxygenation, hyperbaric conditions are necessary which are impractical in a surgical setting. During the 1940s however, doctors began administering N_2_O in combination with a number of other non-volatile anaesthetic agents to allow for lower N_2_O concentrations to be used [1]. Nowadays, nitrous oxide is still used in combination with various anaesthetic agents such as isoflurane and ketamine for anaesthetic sparing, to allow lower concentrations of volatile or non-gaseous anaesthetics to be used [3,4].

Nitrous oxide is also commonly administered in a 50:50 mixture with oxygen to give analgesia during labour as it has no effect on awareness, and can be self-administered by the mother allowing for more personalised pain relief during contractions. In recent years, however, the safety and efficacy of nitrous oxide has been questioned [5,6]. While many studies show adverse effects of nitrous oxide anaesthesia, there is still no general consensus as to whether N_2_O is dangerous enough to warrant discontinuation as an anaesthetic or analgesic [7]. This review article aims to summarise the current evidence for toxicity of nitrous oxide. However, with the limited clinical data presently available on nitrous oxide toxicity it is, as of yet, too soon to draw conclusions.

## 2. Neurotoxicity

It has been documented through a series of clinical studies that nitrous oxide administration is associated with post-operative cardiac problems [6,8]. Further evidence is now mounting which implies nitrous oxide may also cause neurotoxicity. Often, neurological damage may not have overt symptoms and vulnerable patients, such as the elderly, may experience cognitive changes which may go unnoticed. It is often the most vulnerable patients exposed to anaesthetic agents, so one must be sure that the stress of surgery is not exacerbated by the anaesthetic agent which should be providing relief.

Many of the neurotoxic effects of nitrous oxide are dependent on exposure at a certain age or developmental stage. Extensive research indicates that the main periods of vulnerability to nitrous oxide neurotoxicity are during the perinatal period and again in the aged brain. The foundation work has mostly been carried out in rat models but more recent research work has extended into non-human primate models. In rats, perinatal development extends to postnatal day (PND) 7, juvenile rats are classed PND 22–28, adolescence begins around PND 30–35 while adulthood is reached at PND 60 [9]. This demonstrates the rapid development of rats in comparisons to primates and humans.

### 2.1. Perinatal Brain

Rat neurodevelopment is confined to a short period directly after birth, from postnatal day (PND) 0 to PND 7. This roughly translates to a period spanning the third trimester of pregnancy to approximately the 6th month of age in humans [10]. During this period there is a massive increase in programmed cell death as excess neurons are cleared and synapses of remaining neurons are strengthened, known as synaptogenesis. As can be seen in Table 1, rats exposed to N_2_O in combination with other clinical anaesthetics during this period have a consistent, excessive increase in apoptosis in various brain regions, most notably the retrosplenial cortex (RSC) and thalamus [11]. It was also found that these animals have long term impairment of cognitive function [12,13]. It has been shown that at PND 7 in rats there is peak *N*-methyl-d-aspartate (NMDA) receptor expression in the developing brain, which may explain the increased sensitivity to N_2_O [14,15]. This period is approximately equivalent to 20–22 weeks in humans.

**Table 1:** An outline of major *in vivo* studies, regarding the neurotoxicity and mechanisms of nitrous oxide in combination with other anaesthetics, spanning the past 25 years. The studies cover a wide range of ages, anaesthetic concentrations, duration of anaesthesia and even species. Abbreviations: N_2_O—nitrous oxide; iso—isoflurane; PND—postnatal day; mo—month old; BDNF—brain derived neurotrophic factor; RAM—radial arm maze.

Reference	Species	Age	Treatment	Duration	Outcome
[16]	Rat	PND 1–14	75% N_2_O + 0.75% iso + 9 mg/kg midazolam	2, 4, 6 h	Most vulnerable to toxicity at PND7, least vulnerable at PND14 via both extrinsic and intrinsic apoptotic pathways
[17]	Rat	PND 7	75%N_2_O + 0.75% iso ± nociceptive stimulus	6 h	Anaesthesia alone cause neurotoxicity and neurobehavioural deficits, which was exacerbated by nociceptive stimulation during anaesthesia
[18]	Rat	PND 7	75% N_2_O + 0.75% iso + 9 mg/kg midazolam ± melatonin	6 h	Anaesthesia caused neurotoxicity but melatonin decreased neurotoxic damage
[11]	Rat	PND 7	75% N_2_O + 0.75% iso + 9 mg/kg midazolam	2, 4, 6 h	Activates Trk-dependent (thalamus) and Trk-independent, P75NTR dependent (cortex) apoptotic cascade, as well as increasing BDNF
[19]	Rat	6 mo	N_2_O ± ketamine	Not stated	N_2_O toxicity same as ketamine + N_2_O
		18 mo	N_2_O ± ketamine		N_2_O toxicity not as severe as ketamine+N_2_O
[20]	Rat	6 mo	70% N_2_O + 1.2% iso	2 h	Impaired learning and memory in RAM
		20 mo	+ 30% O_2_	2 h	Impaired learning and memory in RAM
[21]	Rat	18 mo	70%N_2_O + 30% O_2_	4 h	Impaired learning and memory in RAM
[22]	Rat	6 mo or 18 mo	70% N_2_O + 1.2% iso	2 h	Aged rats had sustained learning impairment, young rats did not
[23]	Rhesus	PND 5–6	70% N_2_O ± 1% iso	8 h	Alone, no neuronal damage but together caused ↑ caspase-3, Fluoro-Jade-C staining

Using non-human primates, these models can be taken a step closer to clinical relevance, achieving that which would not be ethical or feasible in a human study. No current studies have looked at N_2_O alone, but one study assessed neurotoxicity after a N_2_O/isoflurane mixed anaesthesia protocol in PND 5 or 6 rhesus monkeys [23]. Similar to rodent models, there was widespread cell death in the young monkeys, but interestingly they found a different pattern of distribution in comparison to rodent models. While rodents usually had cell death in the posterior cingulate and retrosplenial cortex (PC-RSC) and thalamus [11,14], this study found widespread apoptosis in the temporal gyrus, hippocampus and frontal cortex [23]. The primate study also found evidence of both necrotic and apoptotic cell death occurring, as opposed to simply apoptotic in the rat. Other studies involving administration of ketamine, another NMDA antagonist anaesthetic, to PND 3–6 rhesus monkeys demonstrated similar patterns of cell death and cognitive dysfunction as rodent models [24,25,26]. These studies also found that by PND 35, the neurotoxic effects of ketamine were no longer present.

Loepke *et al*. [27] undertook a review of all general anaesthetics administered to children in the perinatal period and found a wide range of variability in neurotoxic potential of anaesthetic agents. Nitrous oxide itself had not been subjected to any clinical trials but it was reported that *in utero* or perinatal exposure to N_2_O was correlated with short term neurological problems such as resistance to smiles and increased muscle tone [28]. This indicates that further research into the effects of N_2_O on infants should be undertaken, considering how popular N_2_O is as an induction agent and anaesthetic.

### 2.2. Aged Brain

A study by Noguchi *et al*. [29] compared a range of different aged rats, from PND 20–60, and discovered that at PND 30, MK801 started producing NMDA antagonist toxicity, with PND 60 rats having the highest level of cell death. This indicates that once past the early vulnerable stages after birth, juvenile rats are not as susceptible to the neurotoxic effects of NMDA antagonists, while adolescents are less vulnerable than adults. A number of rodent studies found that N_2_O administration alone or in combination with other anaesthetic agents produced cognitive deficits in aged mice (18–20 months old) [20,21,22]. As shown in Table 1, all studies from this lab used the radial arm maze (RAM) to test cognitive function, which is known to involve hippocampal and cortical memory circuits. One interesting study found that older rats were more susceptible to N_2_O in combination with ketamine than younger (6 month old) rats [19]. They hypothesised that this was due to reduced hepatic function in older rats, which resulted in slower clearance of ketamine from the body. This highlights the fact that N_2_O may not always be reliably compared to ketamine or other non-inhalational anaesthetics, due to their different metabolisation processes.

**Table 2:** Summary of current papers available which study the effects of N_2_O alone, including case studies implicating N_2_O. Abbreviations: N_2_O—nitrous oxide; NMDA—*N*-methyl-d-aspartate; PC-RSC—posterior cingulate-retrosplenial cortex; MS—methionine synthase.

Reference	Species	Age	Treatment	Duration	Outcome
[30]	Rat	Adult	150% N_2_O	Varied	N_2_O acts similar to NMDA antagonists, suggesting it is also an NMDA antagonist
[31]	Rat	Adult	150% N_2_O	1–16 h	Vacuoles present in PC-RSC. Maximal at 3 h+ exposure, persistent after 8 h+ exposure
[32]	Rat	Adult	50% N_2_O	5–80 min	Half-life of hepatic MS inactivation = 5.4 min
	Human		70% N_2_O during surgery	30–290 min	Half-life of hepatic MS inactivation = 46 min
[33]	Human	Adult	Occupational N_2_O exposure	Varied	Increased N_2_O exposure correlates with increased oxidative DNA damage
[34]	Human	Adult	70% N_2_O during surgery	Varied	Increased levels of DNA damage and post-operative wound infection
[35]	Human	3mo	60% N_2_O during surgery (case study)	45 + 270 min	Severe cerebral atrophy, seizures, and apnoea resulting in death
[36]	Human	Adult	N_2_O during dental surgery (case study)	Not stated	Progressive numbness and ataxia, treated successfully with vitamin B_12_ injections

There is also a trend, as seen in Table 2, that in adult rats, high concentrations of N_2_O given under hyperbaric conditions can result in neurotoxicity [31], however due to the fact that these concentrations are unfeasible in a clinical setting, this may not be altogether relevant except to help understand the toxic potential of N_2_O. Together, these results infer that there is a period, beginning in the weeks just after birth, extending until adulthood, where rats appear to have a less severe reaction to NMDA antagonist toxicity, except at clinically irrelevant concentrations. This may be due to changes in the brain during this period, where there is a high level of development but less programmed cell death. In line with these findings, Yon *et al*. [16] showed that between PND7 and PND14, rats become desensitized to the damage induced by an isoflurane/N_2_O/midazolam cocktail, and showed a significant increase in expression of Bcl-XL, an anti-apoptotic protein. Clearly, this warrants further study.

## 3. Molecular Mechanisms of Action

Despite widespread use for many years, the mechanisms by which nitrous oxide achieves its anaesthetic and analgesic properties have still not fully been elucidated. It has been suggested that opioid receptors are responsible for the analgesic properties of nitrous oxide. Research revealed that administration of Nalaxone, an opioid reverse agonist, inhibits the analgesic effects of nitrous oxide [37]. It is well known that there are a range of opioid receptors so it is difficult to pinpoint one specific receptor as being responsible. Work done on the abdominal muscles of mice found that the endogenous ligand for the κ-opioid receptor, dynorphin, may be the mediator of N_2_O antinociception [38,39]. However, both the µ- and ε-opioid receptors were found to have involvement in the rat hot plate test, which involves more peripheral nerves [40]. A study in the guinea-pig brain served to compare binding by nitrous oxide to opioid receptors in the brain and discovered N_2_O acts differently on µ- and κ-opioid receptors. µ-receptors were competitively inhibited by N_2_O while κ-receptors were non-competitively bound [41]. Another mechanism of analgesia appears to be via indirect T-type calcium channel inhibition by N_2_O [42]. This shows there is a high level of variance in how nitrous oxide modulates receptor activity to produce its analgesic effects.

For a drug to have an anaesthetic effect it must decrease excitatory output or increase inhibitory signals to result in a net loss of neuronal activation. In terms of nitrous oxide anaesthesia, the glutamatergic *N*-methyl-d-aspartate (NMDA) receptors have been implicated as a major site of action. NMDA receptors are the natural receptors for endogenous glutamate and are excitatory in nature. In this way nitrous oxide, as an NMDA antagonist, may inhibit excitatory signalling in the CNS. At the simplest neurological level, it was found that NMDA receptors were necessary for the behavioural effects of N_2_O in the nematode *Caenorhabditis elegans*, while volatile anaesthetics such as isoflurane or halothane had another, unspecified mechanism of action [43]. While we cannot directly translate findings in this organism to rodents or humans, NMDA receptors are a highly conserved structure through phyla, allowing for some level of comparison. Jevtovic-Todorovic *et al*. [30] looked at the mechanistic similarities between N_2_O and other NMDA receptor antagonists in an *in vivo* rodent model to discover that N_2_O acted via NMDA receptor antagonism. N_2_O was found to produce neurotoxicity, afford neuroprotection, and induce blockade of NMDA currents, as well as work in the same age-dependent manner as other NMDA receptor antagonists such as MK801. It also provides the same sort of dissociative anaesthesia as ketamine, an NMDA receptor antagonist, overall suggesting that N_2_O likely works through this receptor.

Further work has revealed that N_2_O also has some actions on the two-pore domain TREK-1 potassium channel [44]. This channel functions as a leak channel to release potassium from the cell, stabilising the resting membrane potential in neurons [45]. Previously, it has been shown that TREK-1 channels are important for anaesthesia and knockout mice for the channel are resistant to volatile anaesthetics [46]. TREK-1 has also been found to be important in various types of pain perception [47]. This ion channel could then be a factor in both the anaesthetic and analgesic actions of N_2_O.

## 4. Mechanisms of Neurotoxicity

There are various mechanisms which are responsible for its neurotoxic effects, such as NMDA antagonism, enzyme inhibition and alteration of cerebral blood flow. Different brain conditions have different vulnerabilities to each form of toxicity, with neonatal brains more susceptible to NMDA antagonism, vitamin B_12_ deficient patients more prone to homocysteine mediated problems and the damaged brain often more vulnerable to changes in cerebral blood flow. Because of this, there is a wide range of patients to whom N_2_O may have some form of toxicity, with different groups being at greater risks than others. In this way it is extremely important to understand all the needs of a patient before giving nitrous oxide. The danger arises when nitrous oxide is given during dental procedures or as emergency analgesia, e.g., *en route* to hospital, where underlying problems such as vitamin B_12_ deficiencies may be undetected. One case study details a patient who presented with weakness in her lower limbs as well as peripheral numbness [36]. MRI scans showed abnormalities on the cervical spinal cord consistent with small lesions. The patient was found to be deficient in vitamin B_12_ and had been exposed to nitrous oxide for dental surgeries 2–3 months previously. Following 10 months of vitamin B_12_ injections the symptoms had abated, yet this could have been avoided altogether if nitrous oxide had been avoided for this patient. This highlights the need to fully elucidate nitrous oxide mechanisms of toxicity, so that clinicians can make informed decisions regarding N_2_O use. This case is reflected in further case reports involving patients with no prior N_2_O abuse experiencing myelopathies following N_2_O anaesthesia [48], as well as patients with a history of N_2_O abuse [49,50,51,52].

### 4.1. NMDA Antagonism

NMDA receptors are excitatory receptors in the body which respond to the endogenous agonist glutamate. NMDA antagonists are known to have both protective and toxic effects depending on their activation. As glutamate is an excitatory neuromodulator, excessive release, for example after traumatic brain injury, can lead to excitotoxicity due to high influx of Ca^2+^ into neurons. In this way, NMDA antagonists can provide protection against excitotoxic damage [30,53]. This was shown in a rat model of middle cerebral artery occlusion (MCAO), where 75% N_2_O provided a reduction in cortical, but not striatal, infarcts [54]. This was associated with increased performance in motor coordination tasks compared to MCAO animals with no treatment [55]. While this might suggest some use for N_2_O as a treatment for stroke due to its NMDA antagonist features, it has also been shown that N_2_O has the ability to inactivate tissue plasminogen activator (tPA), as well as increase haemorhage and blood-brain barrier dysfunction [56]. This would preclude its use for stroke as the NMDA antagonist benefits are outweighed by the negative effects.

If administered to the naïve brain, however, N_2_O has the ability to cause neurotoxicity itself. N_2_O has been shown to induce cell death in neurons after prolonged exposure, and shorter term exposure also leads to a more reversible vacuolisation [30,31]. Another potent NMDA antagonist, MK-801 [57], like nitrous oxide, leads to antagonism and thus reduction of signal from excitatory glutamatergic neurons. Despite showing anti-convulsive actions [58], it has not been introduced clinically due to the finding it can form lesions in the brain [59]. It has also been found to alter the structure and function of hippocampal synapses [60]. Jevtovic-Todorovic *et al*. [30] used MK-801 in a comparative study to investigate the possibility that N_2_O was a NMDA antagonist, with N_2_O showing identical physiological outcomes to MK-801. They found that both drugs induce a similar age-dependent toxicity in older rats. They also discovered that administration of GABAergic or muscarinic agents was successful in reversing the vacuolisation of neurons after N_2_O or MK-801 administration [30]. N_2_O is not as potent an antagonist as MK-801 but there appear to be numerous similarities between the two drugs in terms of toxicity. Similar to N_2_O, another NMDA receptor antagonist, ketamine, is often used as an anaesthetic agent. Ketamine is also coming under scrutiny since it was discovered that, like N_2_O, it has the ability to induce reversible or irreversible vacuolisation of neurons [19,25]. Ketamine has also been implicated in causing neuronal cell death by increasing NMDA NR1 subunit expression, leading to increased cytosolic calcium and increased cell death. However, this was after prolonged (24 h) expression and there is little evidence of this mechanism being involved in N_2_O neurotoxicity.

The neurotoxic actions of NMDA antagonists have been attributed to modulation of GABAergic inhibition of various neuronal pathways. The PC/RSC has been associated with learning and memory, as well as pain and awareness. Studies have found that NMDA antagonist administration can result in increased acetylcholine (ACh) release in the PC/RSC as well as the septohippocampal pathway, also involved in learning and memory [61,62]. Normally, NMDA receptors on GABAergic neurons act as an upregulating mechanism to ensure constant inhibitory GABA release. GABA acts upon receptors on cholinergic neurons in the PC/RSC such that ACh release is tonically inhibited. NMDA antagonists release this GABAergic inhibition of cholinergic neurons, allowing ACh release for as long as the NMDA receptor is antagonised. Both studies suggested that NMDA antagonists act not at the region where ACh release is recorded, but instead at some separate site, with GABAergic projections between both sites. In the case of the PC/RSC this was shown to be the basal ganglia [62], while for the hippocampus it appears to be the medial septum. These findings have been extended to show increased ACh release in the cerebral cortex of rats exposed for 1 h with 75% N_2_O [63]. The area postrema is one of the major centres involved in emesis and can be stimulated by acetylcholine [64]. This increased cholinergic output has been postulated to underlie the increased nausea and vomiting often accompanying N_2_O administration. It has yet to be studied if N_2_O can have similar effects on other pathways.

The group led by John Olney [65], who has carried out a mass of work in this field, has referred to this NMDA hypofunction as a cause of complex excitotoxicity. Antagonism of NMDA receptors on GABAergic neurons can release other pathways from the inhibitory control of GABA. These other pathways are usually excitatory in nature, such as the cholinergic pathway investigated above [62]. This excitatory disinhibition has now been implicated in a range of disorders such as schizophrenia and Alzheimer’s disease [66,67,68,69]. While N_2_O most likely does not have as severe an effect as MK801 or ketamine due to its shorter duration of action, it is nevertheless important to consider how these changes in learning and memory centres may affect the very young or old brain.

### 4.2. Homocysteine Imbalance

One side effect of N_2_O which may mediate its toxic effects is indirect inactivation of methionine synthase, an important enzyme in the remethylation pathway converting homocysteine to methionine. Nitrous oxide irreversibly binds to the cobalt atom in vitamin B_12_, also known as cobalamin, via mechanisms which are not well understood. This leads to oxidation of the enzyme [70], eventually causing inactivation of vitamin B_12_. Vitamin B_12_ is an essential cofactor for methionine synthase so inactivation leads to a loss of function of the enzyme. In the normal methionine cycle, methionine is converted to homocysteine (Hcy) via the intermediary molecules *S*-adenosyl-methionine (SAM) and *S*-adenosyl-homocysteine (SAH). From here, homocysteine can either be irreversibly converted to cystathione (eventually becoming glutathione) via the trans-sulfuration pathway, or reversibly converted back to methionine by methionine synthase. Homocysteine, a sulphur-containing amino acid, does not appear to have any inherent function in the body except as a part of this methionine pathway. However, it is known to have various toxic effects in the body so any accumulation can be detrimental. A randomised double-blind study into the effects of anaesthesia post-surgery found that N_2_O administration was associated with higher rates of heart attack, even with patients having non-cardiac surgeries [8]. Homocysteine has been associated with a high rate of cardiac problems [71,72] and patients are found to have elevated homocysteine levels post-surgery [73,74]. This cardiovascular dysfunction appears to be regulated by increasing coagulation and endothelial adhesion, promoting atherosclerosis, as well as altering vascular responses to certain molecules such as argenine via oxidative mechanisms [75].

**Figure 1:**
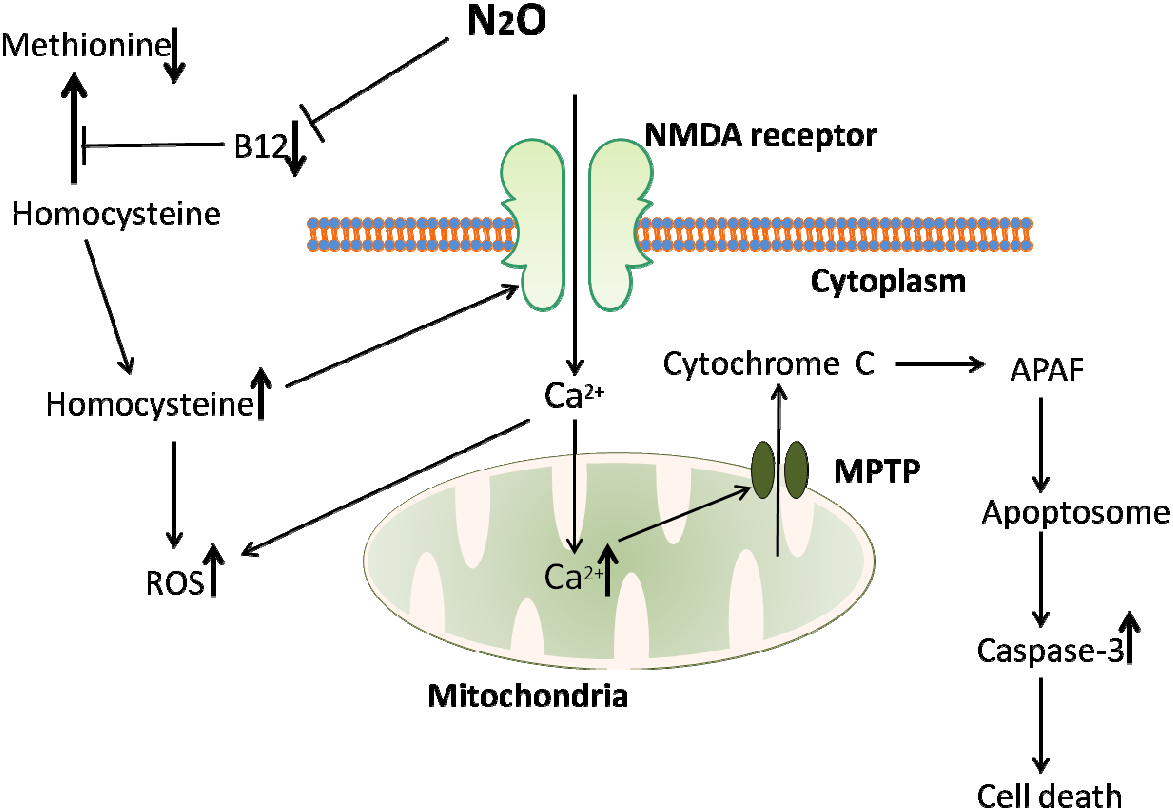
An overview of the homocysteine-mediated pathway of cell death induced by N_2_O exposure. N_2_O inhibits the action of vitamin B_12_, an essential cofactor in the conversion of homocysteine to methionine. This inhibition of vitamin B_12_ leads to a buildup of homocysteine, a toxic amino acid. Homocysteine is toxic via at least two mechanisms; increasing reactive oxygen species (ROS) leading to eventual apoptotic cell death, and NMDA receptor activation. NMDA receptor activation can lead to an increase in ROS due to an influx of calcium into the cell. While N_2_O is also an NMDA antagonist, it is only effective during the course of anaesthetic exposure, while the rise in homocysteine levels induced by N_2_O lasts for hours or even days, suggesting that homocysteine mediated NMDA activation would play a larger role in cell death than N_2_O antagonism could counteract.

Recent interest in homocysteine has revealed multiple pathways by which it causes neurotoxicity at a cellular and cognitive level. Homocysteine has been shown to act as an agonist on the glutamate binding site on NMDA receptors, having an opposing effect to N_2_O (see Figure 1). While this might suggest that N_2_O may counteract homocysteine excitotoxicity, in reality N_2_O is cleared from the system very quickly following cessation of anaesthesia, while homocysteine is known to stay elevated in humans serum for days. In adolescents, homocysteine levels return to baseline between 12 and 24 h [74], while in adults this post-exposure increase is still high at 24 h [73] and continued elevation has been noted for up to one week [76,77]. Lipton *et al*. [78] also report on the dual actions on the NMDA receptor that homocysteine can have. As well as being an agonist, Hcy can also act as a partial antagonist on the glycine binding site of the NMDA receptor. While this might imply that the two binding sites for Hcy would cancel each other out, one being excitatory and one inhibiting the excitatory potential, the reality is more complex, particularly in a brain injury setting. During brain damage such as stroke or traumatic brain injury, glycine levels in the brain become elevated and will overpower the partial homocysteine binding on the NMDA receptor, leading to an elevated excitatory output. Adding to this the agonistic effect of homocysteine on the glutamate binding site, this achieves an even higher level of excitotoxic damage [78].

It is known that methionine synthase inactivation is very fast in rats as compared with humans, with a half-time in rats of 5.4 min *vs*. 40 min in humans when exposed to 50% N_2_O [32] (See Table 2). However, 40 min is still well within the time frame of human exposure during surgery so this should not be taken lightly. Often, these increases in homocysteine may not reach detrimental levels in normal patients, but patients at high risk for hyperhomocysteinemia (HHcy; classed as Hcy levels > 15 µmol/L) could be severely affected if given N_2_O anaesthesia. There are numerous risk factors for HHcy such as Alzheimer’s disease [79], vitamin B_12_ deficiency [80], MTHFR gene mutation [81], age and gender [82]. One striking example of this is a case report involving a young child (3 months old) with an MTHFR gene mutation, leading to an MTHFR enzyme deficiency [35]. This enzyme is important in the remethylation pathway and deficiencies have been linked to HHcy. This particular patient was administered 60% N_2_O on two occasions during surgery, and within 3 weeks after surgery was admitted to hospital suffering from seizures. Less than 2 months post-surgery the patient had died and was found to have severe lesions in the brain, as well as nerve demyelination. At such a young age it is probable that the brain was extremely sensitive to molecular changes and the rapid and extreme increase in homocysteine levels appears to have been involved in the patient’s death. This again highlights the need for clinicians to be vigilant in ensuring their patients are not at risk if exposed to N_2_O. It also showcases the range of physiological parameters which are important to be aware of before administering N_2_O, which dentists and paramedics, who routinely use N_2_O as an analgesic and anxiolytic, do not normally have access to.

### 4.3. Reactive Oxygen Species and Mitochondrial Dysfunction

A range of molecules involved in apoptotic mechanisms in neurons have been found associated with increased homocysteine levels. One of homocysteine’s main mechanisms of cellular damage is oxidative stress, which involves the formation of reactive oxygen species (ROS). ROS are strongly involved in apoptosis and cell death so any increase in levels will be detrimental. It was discovered that NMDA receptor activation leads to production of O_2_^•−^ free radicals in cerebellar granular cells [83], peroxyinitryte (ONOO^−^) in the midbrain [84] and various ROS in the forebrain [85,86]. Since homocysteine can act as an NMDA agonist this may cause increases in ROS. Increased intracellular Ca^2+^ following NMDA receptor activation may account for the increased ROS, whilst ROS can themselves cause a rise in intracellular Ca^2+^ [87,88]. As seen in Figure 1, this increased Ca^2+^ can lead to disturbances in mitochondrial function, resulting in the production of ROS [85,89]. This mitochondrial dysfunction may be a major pathway involved in homocysteine mediated neurotoxicity. It is interesting to note that oxidative stress and mitochondrial ROS formation play a role in Alzheimer’s disease (AD) pathogenesis [90], and it has been shown that high plasma homocysteine is a reliable biomarker for AD [91], although there is no clear consensus as to any causal relationship [92]. AD treatments which act as NMDA antagonists (e.g., memantine) have been shown to reduce homocysteine mediated neurodegeneration [93]. This implies a common underlying mechanism between the two and highlights the damage that can result from homocysteine overload in the brain.

Increased intra-mitochondrial Ca^2+^ also induces formation of mitochondrial permeability transition pores (MPTP), which allows release of cytochrome C from the mitochondria. Cytochrome C can then bind with APAF (Apoptotic Protease Activating Factor) to form an apoptosome, leading to downstream activation of caspase 3, resulting in apoptosis and cell death [94]. It has been shown that the vacuolisation resulting from N_2_O exposure is in fact massive swelling of mitochondria [31]. Drugs increasing mitochondrial membrane stability have been shown to be protective against the neurotoxic effects of N_2_O when combined with midazolam and isoflurane [95]. This membrane stabilisation was associated with improved cognition in rats tested [95].

### 4.4. In Combination with Other Anaesthetics

While N_2_O induced anaesthesia may not show convincing evidence of danger to some, it is also prudent to assess the toxicity of N_2_O in combination with clinically relevant anaesthetic agents to more closely mimic real world scenarios. There are a series of papers which combine N_2_O with isoflurane which consistently show an increase in neuroapoptosis when the two are combined over either agent alone [17,20,96]. These findings have even been replicated in a non-human primate model, the rhesus monkey [23]. It appears that this neurotoxicity is correlated with age; younger animals are susceptible to increased neurodegeneration with isoflurane addition, while adults are less prone to neuronal damage [18,23]. This may be related to the dual function of GABAergic neurons. In young animals, GABAergic neurons are excitatory in nature for a short period postpartum, while in older animals they take on their normal inhibitory function [97]. This may mean that alongside N_2_O induced excitotoxicity, isoflurane, a GABA receptor agonist, can induce extra excitotoxicity, while in adults isoflurane may counteract the excitotoxicity. This excitatory GABAergic action is also found in humans in the few weeks after birth [98], which would suggest that this same enhancement of N_2_O excitotoxicity by isoflurane or any GABA agonist could be present in humans.

## 5. Strategies to Minimize Toxicity of N_2_O

The primary concern in medicine is to cause no harm; therefore it would not be possible to perform procedures without anaesthesia. The stress and damage caused by this would be greater than any deleterious side effects from N_2_O anaesthesia. However, although N_2_O has been used for over a century, it should not be excluded from examination, and if similar or better alternatives are available, they should perhaps be utilised.

A number of possible adjuncts have been put forward. Xenon, another gaseous anaesthetic agent, has been found to be neuroprotective in comparison with other anaesthetics, including N_2_O [13] and has already begun clinical trials for neonates at risk for hypoxic brain damage (CoolXenon2—ISRCTN75602528; Toby Xe—ISRCTN08886155). Melatonin also shows neuroprotective promise when combined with anaesthetics [18]. This may be even more relevant for N_2_O due to the proven effect of melatonin in decreasing homocysteine mediated neurotoxicity in animal studies [99,100].

In terms of possible replacements, a few studies have looked at remifentanil, a fast acting opioid analgesic. Due to its speed of recovery, it has been suggested as a replacement for N_2_O in neurosurgery, as it does not adversely affect cerebral blood flow, unlike N_2_O [101]. Remifentanil has also been suggested as a labour analgesic agent if administered intravenously [102].

## 6. Conclusions

At the moment, it is premature to suggest that N_2_O should be discontinued as an anaesthetic agent. However, the growing body of evidence does support the theory that N_2_O has some neurotoxic effects and these results should not be taken lightly. Nitrous oxide is regularly used for neonatal surgery and, as shown, this is a high risk period for neurodevelopment. It is difficult to assess the long term cognitive outcomes in humans, but rat studies suggest long term developmental issues such as memory impairment. Nitrous oxide is also often used in elderly or brain damaged patients and it is clear from numerous studies, such as the ENIGMA trial, that N_2_O is not as harmless as some might believe. It is important that further molecular work be carried out to determine the pathways by which N_2_O has its toxic effects, as these pathways may reveal areas for drug development to replace or work alongside N_2_O to mitigate its neurotoxic effects. It would also be advisable to carry out further studies on non-human primates to determine any differences between rodent studies. At the moment, from rodent studies, we can only make educated assumptions on what might occur in humans. Non-human primates can help bridge this gap in knowledge without compromising patient safety in clinical trials.
